# Analysis of predictive and prognostic factors in 290 patients treated with oncolytic adenoviruses

**DOI:** 10.1186/2051-1426-3-S2-P344

**Published:** 2015-11-04

**Authors:** Kristian Taipale, Ilkka Liikanen, Anniina Koski, Anna Kanerva, Minna Oksanen, Akseli Hemminki

**Affiliations:** 1 Cancer Gene Therapy Group / University of Helsinki, Helsinki, Finland; 2 Cancer Gene Therapy Group, Medicum, Haartman Institute, University of Helsinki, Helsinki, Finland; 3 TILT Biotherapeutics Ltd, Helsinki, Finland

## Background

After years of development, oncolytic viruses are nearing a breakthrough into clinical use. In order to facilitate the implementation of this new form of immunotherapy, all existing clinical experience must be utilized to optimize treatment strategies, improve patient selection and develop even more effective oncolytic therapeutics.

Here we report clinical factors that affected the prognosis and predicted treatment efficacy in 290 patients treated with oncolytic adenoviruses. In these analyses, survival and efficacy determined by radiological response criteria were used as dependent variables in multi-parameter regression models. In prognostic models measuring hazard ratio (HR) for cancer mortality, tumor type and virus arming were found to affect the prognosis of the patients significantly. Gynecological tumors were notably linked to better prognosis (HR 0.477, p=0,004). Patients treated with viruses coding for GMCSF (HR 0.549, p=0.003) or GMCSF-coding viruses and CD40L-coding viruses (HR 0.439, p=0.023) were more likely to survive longer. In the predictive models the administration of the virus was discovered to independently influence the efficacy of the treatment. Patients who received most of the treatments as intraperitoneal injections were more likely to achieve disease control status (OR 3.878, p=0.016). In a small subset of patients (n=13) who received all treatments intravenously the probability of disease control was even higher (OR 8.995, p=0.032).

## Conclusions

These data indicate several options for further development of oncolytic adenovirus therapy. Concentration on sensitive diseases and choosing the right patient subsets for treatment, as well as finding optimal administration schemes, are key steps in improving the therapeutic efficacy of adenoviruses. The results suggest that evaluating the immunological status of the patients before oncolytic virotherapy can offer important prognostic information. In order to be translated to the field of personalized oncology, this concept requires still further investigation.

